# Biosurfactant as a Promoter of Methane Hydrate Formation: Thermodynamic and Kinetic Studies

**DOI:** 10.1038/srep20893

**Published:** 2016-02-12

**Authors:** Amit Arora, Swaranjit Singh Cameotra, Rajnish Kumar, Chandrajit Balomajumder, Anil Kumar Singh, B. Santhakumari, Pushpendra Kumar, Sukumar Laik

**Affiliations:** 1 Department of Chemical Engineering, Indian Institute of Technology, Roorkee, India; 2 Institute of Microbial Technology, Chandigarh, India; 3 Chemical Engineering and Process Development Division, National Chemical Laboratory, Pune, India; 4 Centre For Material Characterization, National Chemical Laboratory, Pune, India; 5 Keshav Dev Malviya Institute of Petroleum Exploration, Oil and Natural Gas Corporation, Dehradun, India; 6 Department of Petroleum Engineering, Indian School of Mines, Dhanbad, India

## Abstract

Natural gas hydrates (NGHs) are solid non-stoichiometric compounds often regarded as a next generation energy source. Successful commercialization of NGH is curtailed by lack of efficient and safe technology for generation, dissociation, storage and transportation. The present work studied the influence of environment compatible biosurfactant on gas hydrate formation. Biosurfactant was produced by *Pseudomonas aeruginosa* strain A11 and was characterized as rhamnolipids. Purified rhamnolipids reduced the surface tension of water from 72 mN/m to 36 mN/m with Critical Micelle Concentration (CMC) of 70 mg/l. Use of 1000 ppm rhamnolipids solution in C type silica gel bed system increased methane hydrate formation rate by 42.97% and reduced the induction time of hydrate formation by 22.63% as compared to water saturated C type silica gel. Presence of rhamnolipids also shifted methane hydrate formation temperature to higher values relative to the system without biosurfactant. Results from thermodynamic and kinetic studies suggest that rhamnolipids can be applied as environment friendly methane hydrate promoter.

The ever increasing energy demand and depletion of fossil fuel reservoirs have forced researchers to investigate for alternative fuel resources. In recent past natural gas hydrates (NGHs) have been highlighted as a potential next generation energy source^1^. NGHs are non-stoichiometric crystalline ice like structures formed by water and natural gases (like methane, ethane, propane, carbon dioxide and hydrogen sulphide) at high pressure and low temperature^2^. Enormous amount of untapped NGHs are distributed in permafrost regions and in deep sea sediments^3^,^4^. Successful commercialization of NGHs would require efficient and safe technology for their generation, dissociation, storage and transportation.

Gas hydrate formation in a quiescent pure water-gas system involves clustering of water molecules by hydrogen bonding in liquid phase and subsequently occluding gas until a cluster of critical concentration and size is formed. This determines the critical nuclei for hydrate formation. After induction depending upon the system condition, agglomeration of nuclei takes place at water-gas interface resulting in the formation of thin hydrate film on the surface. The thin hydrate film present on surface isolates the bulk water from gas thereby drastically slowing the rate of hydrate formation.

Improving the interfacial interaction between gas-water phases can improve gas hydrate storage and generation. A number of methods have been reported to increase the interfacial contact between systems including mechanical agitation and use of chemical additives^5^,^6^. Mechanical agitation is energy intensive process thus use of chemical additives are preferred. Synthetic surfactants as a chemical additive can enhance hydrate formation rate by increasing gas solubility, supporting micelle formation and providing the nucleation sites for hydrate formation^5^,^6^,^7^,^8^,^9^,^10^. Enhancing the rate of hydrate formation by surfactants can have tremendous influence on commercialization prospect as this can facilitate the conversion of natural gas into solid hydrates useful for storage and transportation of solid NGHs. Use of synthetic surfactant such as sodium dodeccyl sulfate (SDS), sodium tetradecyl sulfate (STS), sodium hexadecyl sulfate (SHS) in natural environment for enhancing NGHs generation could be a cause of concern as they have been reported to possess toxic effects for living organisms^11^,^12^.

Desire to have environment compatible surfactants has propelled search for substitutes of biological origin. Substituting synthetic surfactants with surface active agents of biological origin can provide environment friendly means for enhanced NGHs generation. Surface active agents of biological origin are commonly referred as biosurfactant and often considered better than synthetic counterparts for their low toxicity, environment friendly nature and stability at extreme conditions like temperature, pH, salinity^12^. Very few studies pertaining to influence of biosurfactants on NGHs formation has restricted our knowledge and thus, limiting the potential application of biosurfactants in gas hydrate generation, storage and transportation^13^,^14^,^15^.

The objective of this study is to explore the feasibility of using glycolipids type biosurfactant as methane hydrate generation promoter. Study compares methane hydrate formation in the quiescent water and fixed bed system of water saturated C type silica gel in the presence of different concentration of biosurfactants. The thermodynamics and kinetics of gas hydrate formation was studied to have a better understanding of the process. The study will also help in understanding the role of microbial secondary metabolites on methane hydrate generation at natural sites.

## Results

### Biosurfactant producing microorganism

Glycolipid type biosurfactant used in the present study was produced by *Pseudomonas aeruginosa* strain A11. Earlier strain A11 has been reported as plant-growth promoting (PGP) and multi-metal-resistant (MMR) bacterium capable of producing rhamnolipids while growing on glycerol supplemented minimal salt medium (MSM)^16^. In the present study, growth of strain A11 at 30 °C with agitation of 200 rpm was accompanied by reduction in surface tension of growth medium suggesting production of biosurfactant. Maximum decrease in the surface tension was observed after 36 h of growth when strain A11 was in mid-log phase. Strain A11 decrease surface tension of growth medium from 68.4 ± 0.1 mN/m to 31.9 ± 0.1 mN/m and produced 5020.4 ± 9 .14 mg/l biosurfactant after 72 hours of growth. Specific growth rate (μ) was observed to be 22.2 ± 1.5 mg/l h while specific biosurfactant production rate was 3.14 ± 0.2.

### Characterization of Biosurfactant produced by Strain A11

**Figure 1:**
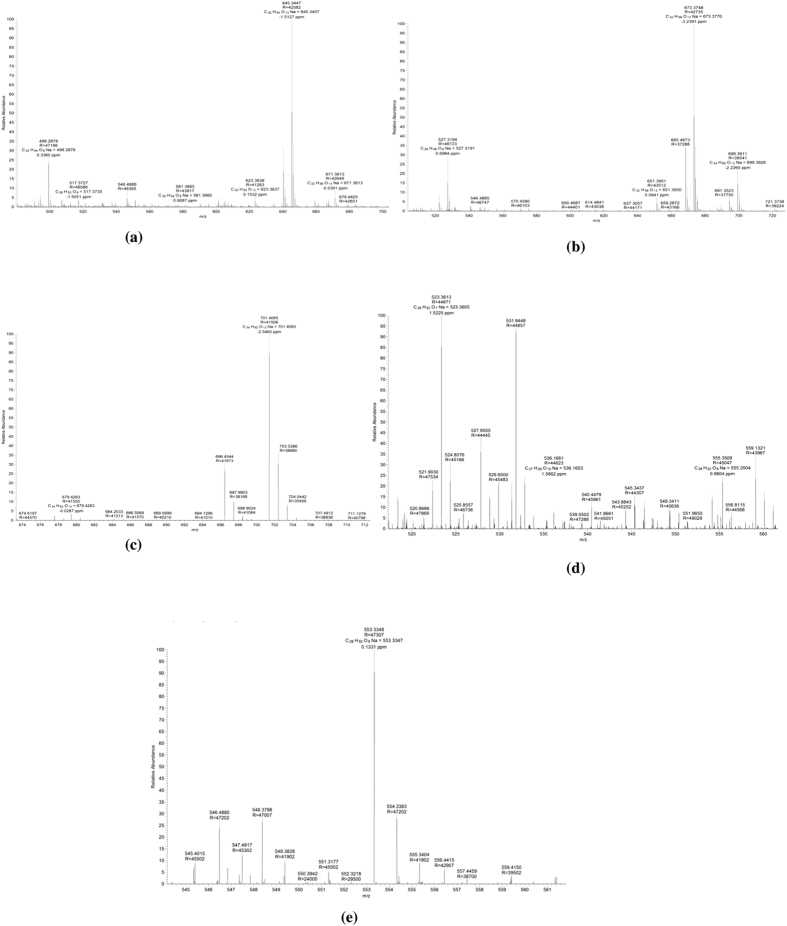
Liquid chromatography-mass spectra of purified rhamnolipid produced by *Pseudomonas aeruginosa* A11 while growing in glycerol supplemented MSM containing TES.

Liquid Chromatography-Mass Spectrometry (LC-MS) mass spectra of purified biosurfactant suggested strain A11 to produce rhamnolipids as a mixture of congeners and homologues Fig. 1a–e. Most abundant rhamnolipids congener observed was dirhamnolipids (RhaRhaC_10_C_10_; α-L-rhamnopyranosyl -α-L-rhamnopyranosyl -β-hydroxydecanoyl -β-hydroxydecanoate) consisting of lipid chain length of C-10. Most abundant monorhamnolipid congener was RhaC_10_C_10_ (L-rhamnosyl -β -hydroxydecanoyl- β -hydroxydecanoate). Interestingly, olefinic rhamnolipids RhaC_22_, RhaC_12_C_10_/RhaC_10_C_12_, RhaRhaC_10_C_10_ and RhaRhaC_12_C_10_/RhaRhaC_10_C_12_ were also observed in the chromatogram. Rarely reported long chain monorhamnolipid congener RhaC_22_ was observed at m/z ratio of 523.36. Table 1 summarizes the list of rhamnolipid congeners with molecular formula, molecular weight and relative abundance in the biosurfactant produced by strain A11.

### Surface Tension, Critical Micelle Concentration (CMC), Stability and Charge on Biosurfactant

Purified biosurfactant from strain A11 reduced the surface tension of water from 72 mN/m to 36 mN/m with CMC of 70 mg/l Supplementary Fig. 1. Interestingly, CMC of biosurfactant from strain A11 did not changed notably even after exposure to extreme temperature range of −20 °C–40 °C and in aqueous solution of pH range 7 to 10 Supplementary Fig. 2. Surface tension reducing ability of rhamnolipids remained intact even after exposure to extreme temperature range of −20 °C–40 °C and in aqueous solution of pH range 7 to 10 Supplementary Fig. 3.

Aqueous solution of rhamnolipids gave a negative zeta potential suggesting it to be anionic surfactant. The zeta potential varied in the range of −26.2 mV to −35.1 mV depending upon rhamnolipids concentration. There was gradual increase in zeta potential till CMC was reached after CMC the zeta potential became stable at −35.1 mV.

### Pore-volume, Pore-diameter and Surface Area of C type silica gel

Brunauer–Emmett–Teller (BET) surface area analysis of C type silica showed that BET surface area of silica used in the study was 540.06 m^2^/g, with the specific pore volume 0.9 cm^3^/g and pore diameter of 49.1A°.

### Methane Hydrate formation-dissociation

**Figure 2:**
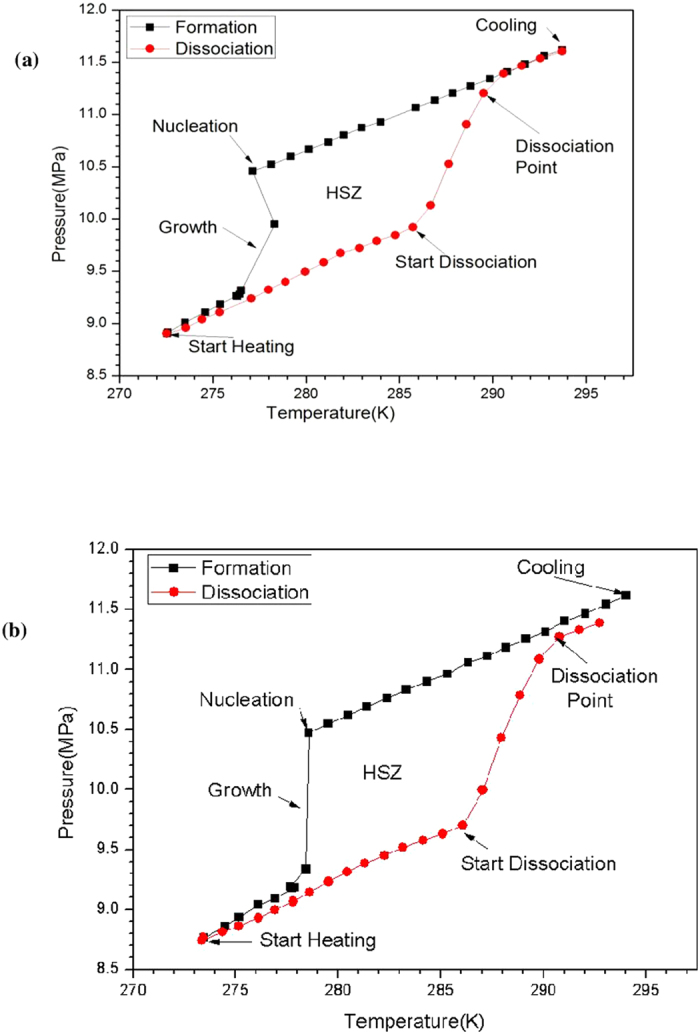
Temperature and pressure profile of methane hydrate formation and dissociation in presence of (**a**) C type silica gel (**b**) C type silica gel containing 1000 ppm rhamnolipid. Values are mean of the results from three individual experiments.

The C type silica gel saturated with distilled water was pressurized with hydrate forming gas and the setup was gradually cooled down which was accompanied by initial gradual decrease in pressure followed by sharp drop in the pressure of 1.14 MPa Fig. 2a. Sudden drop in pressure was observed at 277.16 K along with increase in temperature signifying it to be a nucleation point. After 271.15 K, no significant drop in pressure was observed indicating the completion of hydrate formation. During the dissociation stage gas hydrates were decomposed by thermal stimulation; increased temperature resulted in gradual increase in pressure followed by sudden rise of pressure. Sudden increase in pressure was observed at 285.72 K signifying the start of gas hydrate dissociation Fig. 2a. Dissociation point signifying the complete dissociation of hydrate was observed at 289.52 K.

To observe the effect of rhamnolipids on methane hydrate formation and dissociation, experiment were performed with two different concentrations of surfactant namely 100 ppm and 1000 ppm of rhamnolipids. In the presence of 1000 ppm of rhamnolipid nucleation point got shifted to higher temperature and was observed at 278.59 K Fig. 2b. Rhamnolipids also exhibited influence on gas hydrate dissociation as decomposition started at 286.09 K and complete dissociation i.e. dissociation point was observed at 290.79 K. However, lower rhamnolipids concentration (100 ppm) did not exhibit any note worthy change in the nucleation and dissociation temperature.

A set of experiments were also conducted in the quiescent water system for comparing the influence of chemical additives. As expected methane hydrate formation was extremely slow in a quiescent system at hydrate forming conditions. Hydrate formation was observed at lower nucleation temperature (271.73 K) and nucleation pressure (10.09 MPa) with relatively minimum pressure drop of 0.64 MPa.

**Figure 3:**
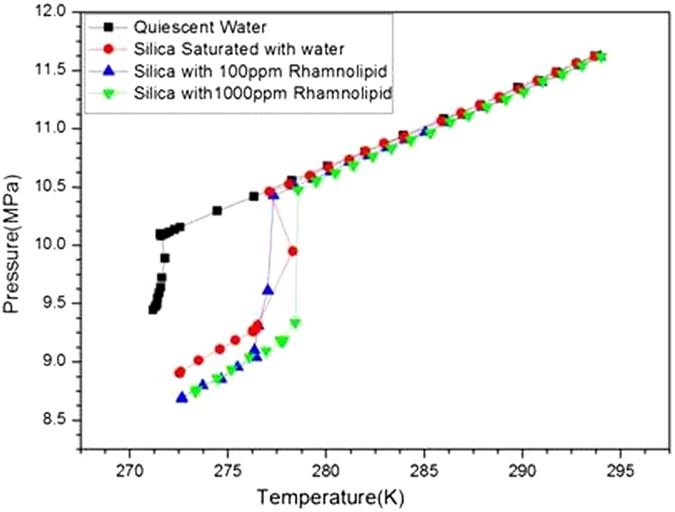
Comparison of methane hydrate formation parameters. Values are mean of the results from three individual experiments.

**Figure 4:**
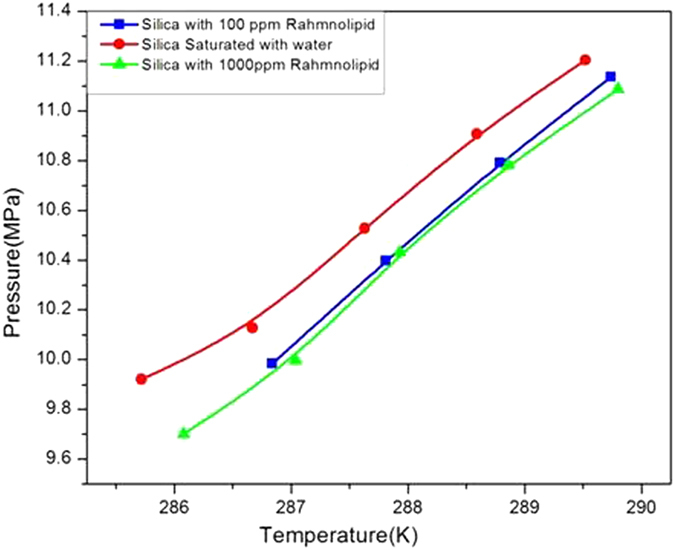
Comparison of phase equilibrium parameters. Values are mean of the results from three individual experiments.

Figure 3 exhibits the hydrate formation temperature under different combinations used in the study. As evident from the figure the nucleation temperature for C type silica was 277.16 K and it almost coincided with the nucleation temperature (277.37 K) of 100 ppm rhamnolipid containing silica gel. However, maximum shift was observed in the presence of C type silica gel saturated with distilled water containing 1000 ppm of rhamnolipids. Figure 4 shows phase equilibrium of methane hydrate at different concentration of rhamnolipids. As evedient from the figure the phase equilibrium curves got shifted to high temperature region in presene of water-silica gel system containing rhamnolipid as compared to the water-silica gel system without rhamnolipids. However, no significant shift of phase equilibrium curve was observed by varing the concentration of rhamnolipid.

### Dissociation enthalpy of methane hydrate

The dissociation enthalpy data of NGHs is very vital for establishing a production scheme. The dissociation enthalpies of methane hydrates in the presence of C type silica gel saturated with distilled water containing different concentration of rhamnolipid were calculated via Clausius-Clapeyron equation and shown in Table 2 based upon the measured phase equilibrium data. No significant difference was observed in the value of enthalpy of dissociation in the presence and absence of rhamnolipid, suggesting that the heat required for dissociating methane hydrate in presence and absence of rhamnolipid is almost similar. The enthalpy of dissociation of methane hydrates in the C type silica gel without rhamnolipid was in the range of 11.8 kj/mol to 18.0 kj/mol. Under that influence of rhamnolipids enthalpy of dissociation was observed in the range of 17.2 kj/mol to 23.0 kj/mol.

### Kinetics of methane hydrate formation

**Figure 5:**
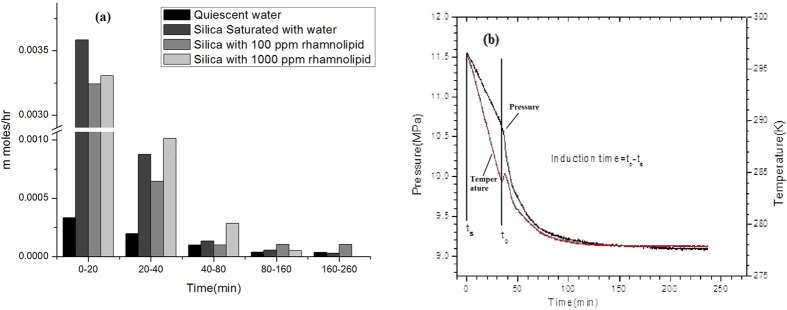
(**a**) Rate of methane hydrate formation for various test samples. (**b)** Induction time for methane hydrate formation in C type silica gel containing 1000 ppm rhamnolipids. Values are mean of the results from three individual experiments.

As evident from Fig. 5a C type silica gel saturated with water and rhamnolipids solution accelerated the rate of hydrate formation as compared to quiescent water system. For better visualization of rate of hydrate generation, the results in Fig. 5a is fragmented into different time frames. The rate of methane hydrate generation in 0–20 minutes time frame in C type silica gel saturated with water is better than the C type silica gel saturated with water- rhamnolipid solution. However, after aforementioned time frame presence of rhamnolipids in the system enhanced the rate of hydrate formation.

A certain time is required to initiate hydrate nucleation when hydrate forming components are placed in the suitable temperature and pressure region. The induction time is determined by measuring the time taken by the system to reach the hydrate onset temperature (where detectable volume of hydrate is observed). The induction time of hydrate formation was measured in quiescent water system and in C type silica gel consisting of different concentration of rhamnolipids. During hydrate formation initially there was a gradual decrease in pressure with temperature before hydrate formation started Fig. 5b. Afterwards a sharp peak is seen due to sudden rise in temperature with simultaneously decrease in pressure confirming hydrate formation. The difference of hydrate onset time and the time of start of experiment is the induction time of hydrate formation. The induction time for quiescent water, C type silica gel without presence of surfactant; C type silica gel containing 100 ppm rhamnolipid and C type silica gel containing 1000 ppm rhamnolipid was 196.70 min, 44.24 min, 36.91 min and 34.23 min, respectively. As evident from Fig. 5b addition of rhamnolipids decreased the induction time of hydrate formation significantly as compared to quiescent water and C type silica gel system.

### Moles of methane consumed per mole of water

**Figure 6:**
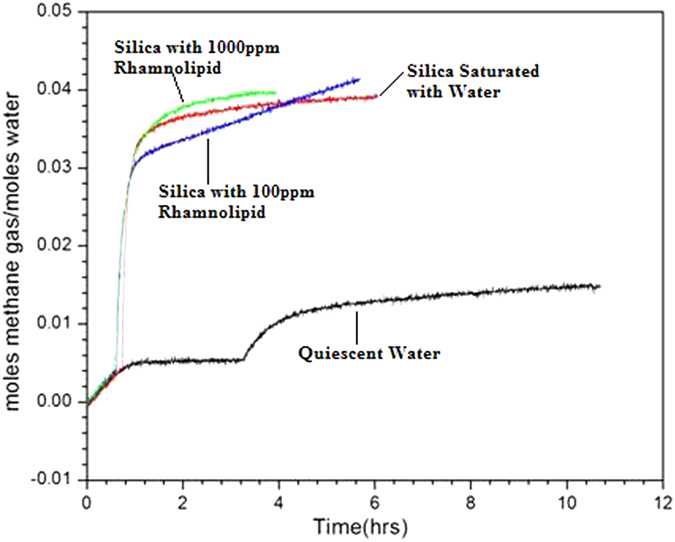
Moles of methane per moles of water consumed while methane hydrate formation. Values are mean of the results from three individual experiments.

Figure 6 shows the gas uptake measurement curve (moles of methane consumed per mole of water in the system) of a methane hydrate formation experiment. The general characteristic of methane uptake curve in quiescent water does not resemble that of hydrate formation in silica system saturated with water or water-rhamnolipids solution. As compared to quiescent water system the number of moles of methane consumed per water molecule was significantly more in silica system. There was nearly 2.6 times more methane gas consumption in silica system saturated with water. Presence of rhamnolipids in the silica system further increased methane gas consumption per water molecule during methane hydrates formation.

## Discussion

Biosurfactant is regarded as a next generation surfactant for its environment friendly nature and versatile potential applications^17^. Use of biosurfactant in NGHs formation can make process environment friendly and more acceptable to environmentalist and policy makers.

In the present study biosurfactant used was obtained from strain A11. Strain A11 produced rhamnolipid while growing on relatively inexpensive carbon substrate glycerol. In current scenario, utilization of glycerol for the production of biotechnologically valuable products is gaining momentum as it is relatively inexpensive^18^. Also the amount of waste glycerol production is increasing year by year through the increasing production of biodiesel and other oleochemicals^19^. The amount of rhamnolipid produced by strain A11 under given condition is more than several others reports^20^,^21^. Previously strain A11 has been reported to produce 4,436.9 mg/l of biosurfactant after 120 h of incubation^16^. However, in the present study yield was increased by adding Trace Element Salts (TES) and increasing carbon source concentration in growth medium. Growth limiting conditions are known to promote rhamnolipids production^17^. Limiting concentration of multivalent ions such as Mg, Ca, K, Na, and trace element salts are known to increase rhamnolipid yield^22^. High yield, efficient purification and low substrate cost can make biosurfactant economically viable for industrial applications. Also considering the environmental damage that synthetic surfactant causes makes biosurfactant form strain A11 economically better choice for enhance NGHs production.

In recent past our knowledge about the rhamnolipid diversity has increased primarily due to use of more sensitive and sophisticated analytical techniques^23^. Changing abiotic factors like growth media and conditions are known to influence rhamnolipids homologous and congeners composition^24^,^25^. Earlier Singh and Cameotra^16^ have reported that strain A11 produced biosurfactant predominantly consisting of dirhamnolipids with single monorhamnolipids congener. However in the present study, with the help of more sophisticated analytical method unrecorded homologous and congeners were also observed. Usually RhaRhaC_10_C_10_ is the most abundant rhamnolipids congener^17^. Literatures suggest that the observed difference between components and content of fatty acids can be attributed to change in culture condition and the analysis techniques^17^,^23^.

The purified rhamnolipid reduced the surface tension of water to 29 mN/m with CMC of 83 mg/L^16^. However, a small change in the surface tension reduction ability and CMC was observed in the present study as compared to the previous report. This may be attributed to change in the composition of the biosurfactant and the content of fatty acid components. Similar changes have also been highlighted earlier^24^.

The CMC is a vital parameter of any surfactant as it signifies the efficiency of surfactants. Lower CMC surfactants are in very much desirable as concentration of surfactant needed for lowering the surface tension is very small. Each component of rhamnolipids mixture contribute differentially towards CMC but due to difficulty in separating the rhamnolipids components in to a single homologue, the individual contribution of each component in CMC has not been elucidated. The CMC of different *Pseudomonas* rhamnolipids varies from 53 to 230 mg/l depending on the ratio and composition of rhamnolipids species^22^. Higher potency of biosurfactant makes it better than several commercial synthetic surfactants. Literature suggests that rhamnolipids is more efficient than most common synthetic surfactants like Sodium Dodecyl Sulphate (SDS), Triton^26^,^27^. Mendes *et al*. (2014) observed rhamnolipids to be more efficient than SDS^28^. At CMC, the SDS reduced the surface tension of water to ~36 mN/m as compared to rhamnolipid that reduced the surface tension to ~27 mN/m. Also the CMC of SDS was 100 time higher than rhamnolipids^28^.

Stability of rhamnolipid at extreme conditions can be attributed to highly stable bond between sugar moiety and lipid chain. Similar observations have been reported by Singh and Cameotra^16^. Mendes *et al*. reported rhamnolipids to be thermoresistant up to a temperature of 80˚C. Rhamnolipids are weak acid and have a pKa of 5.6^29^. CMC of rhamnolipids changes significantly if variation in pH values is brought about below or above 5.6. Sanchez *et al*., (2007)^30^ demonstrated that at pH 7.4 the CMC of dirhamnolipid is 0.110 mM, whereas at pH 4.0 it falls to 0.010 mM. Thus, suggesting that a negatively charged rhamnolipid has a much higher CMC value than the neutral species. However, in present study rhamnolipids solution was challenged to pH of 7 and above. Hence, no significant change in CMC was observed with respect to variation in pH.

Environmental factor such as pH and temperature play a decisive role in influence the competence of rhamnolipids. Generally pH of sea water ranges from 7.5 to 8.4 where as temperature can reach minimum upto subzero levels and maximum upto 36 °C depending upon surrounding conditions. Stability observed under aforementioned condition suggests that rhamnolipid can be successfully used in the marine conditions.

Zeta potential is the potential difference between the dispersion medium and the stationary layer of the fluid attached to the dispersed particle. The zeta potential value relates to the stability of colloidal dispersion. Colloids with high zeta potential are electrically stabilized. Anionic nature of rhamnolipids observed in the present study is in accordance with the earlier report^31^.

Lanoil *et al*. reported direct physical interaction between microbes and gas hydrate^32^. They hypothesized that aforementioned interaction can have important implications for gas hydrate stability, composition, and geochemistry. Members of gammaproteobacteria have been reported in Gulf of Mexico gas hydrates samples^32^. Thus, anticipation that secondary metabolites from gammaproteobacteria like *P. aerugeniosa* may have beneficial effect on gas hydrate formation would not be an exaggeration. It was expected that rhamnolipid, a glycolipids type biosurfactant produced by *P. aerugeniosa* strain A11 can execute beneficial influence on gas hydrate formation by improving interfacial interaction between water and methane.

In present study, for making the NGHs technology environment friendly along with microbial surfactant, environment compatible porous media was also used. Earlier, Linga *et al*. highlighted the need for studying the dynamics of hydrate formation and decomposition in natural porous media as there are very few reports in literature^33^. As compared to quiescent water system rate of hydrate generation was rapid in C silica gel due to better gas-water contact^34^. The C type silica gel has smaller particle size and higher specific surface area as compared type A and type B silica gel, thus has better hydrate conversion ratio^34^. Combination of larger pore diameter and higher surface area enhance the rate of hydrate formation and water to hydrate conversion ratio significantly. In a porous matrix hydrates formation takes place within the pores and in between the interstitial sites. Larger pores enhances the diffusion of gases into the interstitial sites while high surface area allows more contact between the water and gas thus, resulting in better hydrate conversion.

The dissociation enthalpy data of hydrate is very vital for establishing a viable production scheme. Dissociation enthalpies of gas hydrates can be calculated either direct by calorimetric measurement or indirectly via the Clapeyron or Clausius-Clapeyron equation by differentiating of phase equilibrium pressure-temperature data. The system during dissociation is assumed to be in the state of equilibrium, the temperature pressure data of dissociation under this condition can be applied to calculate the enthalpy change of dissociation and association of gas component. Several groups have calculated the enthalpy of formation and dissociation by calorimetric method^35^,^36^. Handa *et al*. reported that calorimetric method for calculation of enthalpy is troublesome because NGHs are stable at low temperatures and high pressures, and for valid outcome sophisticated instrumental setup is required^37^. Although the calorimetric measurement can be considered better than the results obtained from phase equilibrium data, simplicity and handiness of process make it very useful. In present study the values of enthalpy calculated by Clausius-Clapeyron equation are close to that calculated by direct measurement method^35^,^36^. The value of methane hydrate dissociation in present study at 10.4 MPa and 287.8 K is found to be 23.003 kJ/mol and the same reported by Gupta *et al*., (2008)^36^ at 9.8 MPa and 285.6 K is found to be 55.21 kJ/mol^36^.

Information on kinetics of NGHs formation is vital for storage, transportation and effective utilization of NGHs hence, have received considerable attention in the recent past. In present study, the chemical additives significantly increase the rate of hydrate formation as compared to quiescent water system. Comparatively better methane hydrate formation kinetics in C type silica can be attributed to the large surface area and large pore diameter which facilitated the relatively better mass transfer between two phases leading to large pressure drop due to nucleation and growth. Faster growth kinetics at a later period in the presence of surfactants can be attributed to lower interfacial tension allowing better contact between methane and water thus better mass transfer between the two phases.

The induction time in methane hydrate crystallization is an important characteristic of the kinetics studies. Various groups have given different definitions of the induction time. In present study, it was reported as time elapsed between the beginnings of the experiment to the onset of hydrate formation^38^,^39^. A temperature spike observed in present study is due to latent heat released because of gas hydrate formation^40^. During a typical gas uptake measurement, the temperature rises to a maximum value coinciding with hydrate nucleation, however the temperature controlled water bath brings it down to the operating temperature. Release of energy due to hydrogen bond formation during hydrate nucleation is responsible for this sudden rise in reactor cell temperature^41^. Shorten induction time as observed in the presence of silica gel is attributed to higher surface area of C type silica gel. Silica gel allowed a better contact between the gas and water phase resulting in lower induction time.

Replacing energy intensive process of agitation with fixed bed system decreases over all energy requirement and confiscates the requirements for specially designed system. Autoclave used in the present study was earlier used by Saw *et al*. for studying the kinetics of methane hydrate formation and its dissociation in the presence of Tergitol with agitation of 1000 rpm. Interestingly the fixed bed system of C type silica gel used in the present study was found to be better than the combination of agitation and surfactant in terms of induction time of methane hydrate formation.

In present study saturating C type silica with water-rhamnolipids further favored methane hydrate formation as compared to condition when only water was used for preparing silica bed. Presence of rhamnolipids shifted methane hydrate formation temperature to higher value. The equilibrium temperature and pressure of methane hydrate formation shifted to higher and lower values, respectively. The rate of hydrate formation increased as well as the induction time of hydrate formation got reduced in the presence of rhamnolipid suggesting rhamnolipids as methane hydrate promoter.

Earlier few groups have carried studies on the effects of biosurfactants on gas hydrate formations and highlighted them as promoter^13^,^14^,^15^,^42^. Rogers *et al*. reported that addition of commercially available rhamnolipid increased the hydrate formation rate by 96% and decreased the induction time by nearly 60% as compared to the system consisting of seawater, natural gas and sand-clay where surfactant was not used^13^. Recently, Wang *et al*. reported use of clathrates of biological origin for accelerating hydrate formation kinetics^43^. They reported the nucleation temperature in the presence of fungi to be 279K. Present study also reports almost same temperature (278.59K) in silica bed containing 1000 ppm rhamnolipids solution.

In quiescent water system the number of moles of methane per mole of water consumed is minimum mainly because of hydrate layer formation on the water surface subjecting inadequate interaction between the gas-bulk water. On the contrary use of porous C type silica gel in present study increased the gas uptake primarily by providing more surfaces for interaction. Further use of biosurfactant solution with C type silica gel favored the methane consumption by reducing the surface tension of water.

Chemical nature and concentration of surfactant have significant influence on NGHs promoter activity of surfactants. Anionic surfactants are more effective than nonionic and cationic surfactants in enhancing the rate of hydrate formations^44^. Karimi *et al*. studied influence of three synthetic surfactants namely sodium dodecylbenzenesulfonate (SDBS; anionic surfactant), dodecyl trimethyl ammonium bromide (DTAB; cationic surfactant) and TritonX-100 (nonionic surfactant) on ethane hydrate generation. They observed that SDBS are more effective in enhancing the ethane hydrate formation rate at various concentrations. Cationic surfactant DTAB has the opposite effect on the ethane hydrate formation rate, which decreased with increasing DTAB concentration. Nonionic surfactant TritonX-100 also increased the ethane hydrate formation rate but was not as effective as SDBS^45^.

Biosurfactant concentrations above CMC are more effective in fastening the rate of NGHs formation than the concentrations below CMC^13^. The biosurfactant promote the NGHs formation by associating water to hydrophilic head and hydrocarbon gas to hydrophobic tale^15^ and also by “Surfactant micelle hypothesis” suggested by Rogers and his coworkers^13^,^46^,^47^,^48^,^49^. Micelles are considered as colloidal aggregates which are formed by surfactants in solution when the concentration of the surfactant exceeds CMC. The natural inclination of micelles to gather large masses of structured water and hydrocarbon gas at a common site increases their likelihood of promoting hydrate formation. Micelle plays the role of nucleation point which increase the solubility of hydrocarbon gas in the aqueous phase. It leads to the formation of hydrate crystals around the micelle.

The above hypothesis was challenged by Watanabe *et al*.^50^. He proposed that several surfactants including SDS cannot form micelles at a hydrate forming temperature. The lowest temperature at which the micelles can be formed is known as Kraft’s point. The rhamnolipids are in the form of congener consisting of monorhamnolipids and dirhamnolipids. The kraft point of rhamnolipids having more dirhamnolipids is below 0 °C^51^. In the present study the relative abundance of dirhamnolipids is far greater then monorhamnolipids and it can help in reaching the kraft point at hydrate formation conditions. Moreover, Watanabe *et al*.^50^ observation was based on HFC-32 fluorohydrocarbon-gas/water/SDS system at low pressure. The results were then extrapolated to methane/water/SDS system which their group did not evaluate^52^.

Literature suggests that among all the currently known synthetic surfactants, SDS has been widely reported as NGHs promoter^5^,^9^. Relatively better know how of SDS as NGHs promoter makes it as forerunner among the synthetic surfactants for industrial application. However, SDS toxicity cannot be ignored towards living organisms. SDS reaches living organism by food chain, gets accumulated and induce toxic effect by damaging biological macromolecules like protein, lipids, phospholipid membranes and DNA. Substituting SDS with environment compatible anionic rhamnolipids is a good option. Moreover, CMC of rhamnolipids is ~100 times lower than that of SDS thus making former a more efficient surfactant^28^.

## Materials and Methods

All the chemicals and reagents used in the present study are of the highest purity grade available. The C type silica gel were purchased from Merck Specialties Pvt. Limited, Mumbai, India and used without further treatment. Methane with purity of 99.99% was procured from Chemtron Science Laboratory, Mumbai, India. Microbiological growth media were supplied by HiMedia, India. Water used in the study was de-ionized in Milli-Q equipment (Millipore, Bedford, MA) and had a resistivity of 18 MΩ.

### Microorganism and growth medium composition

Biosurfactant producing microorganism *P. aeruginosa* strain A11 was isolated from rhizosphere of wild plant *Parthenium hysterophorus* growing at Dalma Wildlife Sanctuary, Jamshedpur^16^. For biosurfactant production strain A11 was grown in glycerol 4% (v/v) supplemented (MSM). Composition of MSM was slightly modified from earlier reported one by adding 1 ml TES per liter of MSM^16^. TES contained 0.1 g Al(OH)_3_, 0.05 g SnCl_2_·2H_2_O, 0.05 g KI, 0.08 g MnCl_2_·4H_2_O, 0.05 g LiCl, 0.5 g H_3_BO_3_, 0.1 g ZnSO_4_·7H_2_O, 0.1g CoCl_2_·6H_2_O, 0.1 g NiSO_4_·6H_2_O, and 0.05 g BaCl_2_ in 1 l of the solution.

### Biosurfactant production and purification

Biosurfactant production was carried out in Erlenmeyer flasks (2 l) containing 500 ml aliquots of MSM. Optimized conditioned reported by Singh and Cameotra^16^ was followed for biosurfactant production. Biosurfactant purification was performed following method reported by Sanchez, *et al*.^30^ with slight modification. In brief, cell-free supernatant (CFS) was obtained by centrifuging the culture broth at 8,000 × g for 10 min at 4 °C. The CFS was acidified to pH 2 with 6N HCl and incubated overnight at 4 °C. The white precipitate was collected by centrifugation (10,000 x g for 10 min). The precipitate was dissolved in 50 mM NaHCO_3_ buffer (pH 8.6) and again re-precipitated with 6N HCl. From precipitate the biosurfactant was obtained by solvent (2:1 chloroform and methanol) extraction at ambient temperature. Solvent was evaporated under reduced pressure to obtain honey coloured viscous biosurfactant.

Concentrated viscous biosurfactant was dissolved in chloroform (5 mg/ml) and then purified by activated silica-gel 60, column (2 × 40 cm). Column was loaded and washed at a flow rate of ~1 ml/min, using chloroform till neutral lipids were totally eluted. Then column was washed with chloroform/methanol 50:50, and pure methanol to elude the rhamnolipids congeners. The composition of each fraction was analyzed by thin layer chromatography (TLC) on silica gel plates^16^. Fractions containing rhamnolipids were concentrated using rotary evaporator. Concentrated biosurfactant was lyophilized to obtain white powder.

### Determination of Biomass and Biosurfactant Concentration

Cell growth was monitored by measuring the absorbance at 600 nm. For the determining biomass concentration (g/l) calibration curves of dry weight (g/l) verses absorbance at 600 nm was used. Biosurfactant concentration in culture broth was determined by quantifying hydrolysis released rhamnose by orcinol method after ethyl acetate extraction and acid hydrolysis of the samples^53^. Monod equation was used to measure specific growth rate (μ) of strain A11 in MSM^54^.

### Surface tension and Critical micelle concentration (CMC)

Surface tension was determined at 25 °C using a duNouy tensiometer (CSC Scientific Company Inc., USA) based on platinum-iridium ring detachment method. For the calibration of the instrument ultrapure water (72 mN/m) and pure ethanol (22.7 mN/m) was used to make sure accuracy over the entire range of surface tension measurements. Un-inoculated growth medium was used as negative control. The CMC was determined by plotting the surface tension as a function of biosurfactant concentration and point of a sudden change in the surface tension was designated as CMC^16^.

### Zeta potential measurements

The zeta potential measurements were carried out for determining the ionic nature of biosurfactant. Aqueous solution of biosurfactant was prepared in the range of CMC to 5CMC and the solution was subjected to zeta potential measurement at 25 ± 1 °C by Zetasizer (Malvern Instruments Ltd., (UK).

### Liquid Chromatography-Mass Spectrometry (LC-MS)

Purified biosurfactant was characterized by analysing on hybrid Quadrupole-Orbitrap Mass Spectrometer (Q-Exactive, Thermo Fisher Scientific, Austria) coupled to UHPLC system consisting of a LC-pump (Accela), degasser and autosampler. Chromatographic separation was achieved using Hypersil Gold C_18_ (8 μm, 150 × 4.6 mm) reverse phase column. Mobile Phase system consisted of 40% acetonitrile (ACN) and 60% water with 0.1% formic acid for initial four minutes, following next four minutes percentage of ACN was gradually increased from 40% to 90%, and for rest of run (22 minutes) the percentage of mobile phase was maintained at 90% ACN at a constant flow rate of 500 μL/min, at ambient temperature. The auto sampler was set to inject 5 μL of sample with a chromatographic run time of 30 min. The tuning parameters for the MS were set as follows: capillary temperature 320˚C, spray voltage 3.60 kV, heater temperature 350˚C, sheath gas flow rate 45, auxiliary gas flow rate 10 and sweep gas flow rate is 2. All mass spectra were acquired in the full scan positive ionization mode from m/z 400 to 900 m/z. Data acquisition and processing were performed using Thermo Xcalibur Qual browser (Version 2.2).

### Brunauer–Emmett–Teller

BET Nitrogen adsorption-desorption isotherms for C type silica gel were obtained using a conventional volumetric nitrogen adsorption apparatus (Quantachrome Instrument Autosorb1 system). The sample was degassed at 250 ^o^C for 12 h at 0.00133 Pa, prior to the measurements. The sample was cooled to –196 ^o^C using liquid nitrogen and the sorption of nitrogen was carried out at different equilibrium pressures. The specific surface area of the sample was calculated using Brunauer, Emmett, and Teller (BET) method. The pore size distribution was calculated using the Barret- Joyner-Halenda (BJH) pore size model applied to the adsorption branch of the isotherm. The isotherms were classified as the type IV adsorption desorption defined by IUPAC which gives useful information through its hysteresis loop.

### Experimental Set Up and Procedure

Schematic diagram of the autoclave used in present study for hydrate formation is given in supplementary Fig. 4. The gas hydrate formation and dissociation was studied in the mercury free video hydrate cell designed by Vinci Technology, France. Data acquisition was performed by built in software available with the system. The experiments were conducted in quiescent water system and porous C type silica gel saturated (90%) with distilled water containing different concentrations of rhamnolipids (0, 100 and 1000 ppm).

The apparatus consisted of a constant volume hydrate cell having capacity of 250 cm^3^ (diameter = 8 cm and height 2.5 cm) with pressure rating up to 3000 psi. A bed height of 2.5 cm was prepared by using 64.7 g of C type silica and was 90% saturated with 52.41 ml of water. Thermostatic bath was used to control the cell temperature. Cell pressure was measured by pressure transducer.

The test sample was placed in hydrate cell immersed into a temperature controlled bath to maintain a constant temperature. The air in the cell was removed from the cell before pressurizing methane gas in the cell by vacuum pump. Methane gas up to the desired pressure was pressurized in the cell. The same experimental setup was used for carrying hydrate stability zone and kinetics experiments. For conducting the hydrate stability zone experiments the stepwise cooling (1 K/h) of the programmable bath was done and sufficient time was provided to acquire the equilibrium condition at each temperature. A sudden pressure drop and increase in temperature was detected for the hydrate formation. Online video picture were also used for detecting the hydrate formation. Then further temperature was decreased slowly to monitor pressure drop due to growth of hydrate. When insignificant pressure drop was noted suggesting completion hydrate formation, the system was kept to remain at the same temperature for 4 hours to gain a state of equilibrium. After hydrate formation, the heating of the entire cell was done at a rate of 1 K/h.

Kinetics experiments of the same test samples were carried out to measure induction time and rate of hydrate formation after depressurizing of gas hydrate and then placing the cell at the methane hydrate formation conditions observed from the stability experiment.

### Data analysis

The heat of dissociation is measured by the Clausius-Clapeyron equation is as follows:


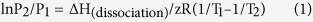


where P_1_ stands for initial pressure, P_2_ stands for final pressure, T_1_ stands for initial temperature, T_2_ stands for final temperature, z stands for compressibility factor for gas, R stands for universal gas constant(R = 8.14 J mol^−1 ^k^−1^) and ΔH (dissociation) KJ mole^−1^ stands for molar enthalpy of dissociation of methane gas hydrate.

The rate of methane hydrate formation for different experimental conditions was assumed to be first-order reaction. A first order reaction is defined by the following Equations 2 and 3:


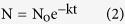



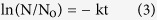


where N stands for total number of moles at time t, N_0_ stands for initial number of moles, k is the rate constant (min^−1^) and t is time in minute. The rate constant (k) of hydrate formation can be obtained from the slope of the curve of ln (N/N_0_) vs time. Hydrate formation rate is calculated by putting the values of slopes i.e. rate constant k in the following equation:


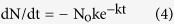


The total number of moles of the gas that were consumed for hydrate formation was calculated by equation


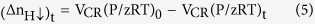



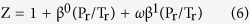



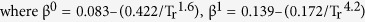



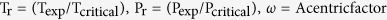


where z is the compressibility factor calculated by Pitzer’s correlation Smith *et al*.^55^ varies as the reaction proceeds which are obtained by the gas uptake data. V_CR_ is the volume of the gas phase in the crystallizer; P and T are pressure and temperature of the crystallizer at corresponding times.

## Additional Information

**How to cite this article**: Arora, A. *et al*. Biosurfactant as a Promoter of Methane Hydrate Formation: Thermodynamic and Kinetic Studies. *Sci. Rep*. **6**, 20893; doi: 10.1038/srep20893 (2016).

## Supplementary Material

Supplementary Information

## Figures and Tables

**Table 1:** Assignment of all rhamnolipid mass peaks obtained by LCMS mass spectrometry of biosurfactant produce by *Pseudomonas aeruginosa* strain A11 while growing on glycerol supplemented MSM containing TES.

Congener	Pseudomolecular mass (m/z)	Retention Time (min.)	Molecular Formula	Relative Abundance (%)
RhaC_10_C_8_/RhaC_8_C_10_	499.28	13.97	C_22_H_44_O_9_	3.4
*RhaC_22_	523.36	23.47	C_28_H_52_0_7_	2.07
RhaC_10_C_10_	527.32	16.05	C_26_H_48_O_9_	9.76
*RhaC_12_C_10_/RhaC_10_C_12_	553.33	17.45	C_28_H_50_O_9_	1.13
RhaC_12_C_10_/RhaC_10_C_12_	555.35	19.42	C_28_H_52_O_9_	0.46
RhaRhaC_10_C_8_/RhaRhaC_8_C_10_	645.34	12.95	C_30_H_54_O_13_	12.16
*RhaRhaC_10_C_10_	671.36	13.88	C_32_H_56_O_13_	1.35
RhaRhaC_10_C_10_	673.37	14.76	C_32_H_58_O_13_	32.12
*RhaRhaC_12_C_10_/RhaRhaC_10_C_12_	699.39	15.94	C_34_H_60_O_13_	7.49
RhaRhaC_12_C_10/_RhaRhaC_10_C_12_	701.4	17.53	C_34_H_62_O_13_	11.83

*Olefinic Rhamnolipid.

**Table 2:** Equilibrium temperature, pressure and dissociation enthalpy calculated by Clausius-Clapeyron equation.

Type of sample	P (dissociation) (MPa)	Ln(P) (MPa)	T (dissociation) (K)	1000/T (k^−1^)	Z factor	ΔH_d_ KJ (mol^−1^)
C-type Silica gel without presence of Rhamnolipid	9.9215	2.2947102	285.7	3.49993	0.8071	–
	10.128	2.3153434	286.6	3.488332	0.80086	11.84493
	10.528	2.3540659	287.6	3.476689	0.7989	16.96316
	10.907	2.3894515	288.5	3.465124	0.79771	18.05239
	11.203	2.4162691	289.5	3.45137	0.79739	17.54297
C-type Silica gel containing 100 ppm Rhamnolipid	9.98361	2.3009447	286.8	3.486264	0.8028	–
	10.39729	2.3415452	287.8	3.474514	0.8007	23.003
	10.7903	2.3786476	288.7	3.462724	0.79931	21.93559
	11.13503	2.410096	289.7	3.45137	0.7986	20.76912
C-type Silica gel containing 1000 ppm Rhamnolipid	9.70092	2.2722207	286.08	3.495526	0.8043	–
	9.9974	2.3023251	287.04	3.483835	0.8044	17.22145
	10.43177	2.344856	287.93	3.473066	0.8007	21.5293
	10.7834	2.3780079	288.86	3.461885	0.7995	20.90218
	11.08677	2.4057525	289.8	3.450656	0.7992	19.77393
