# Supplementary material for: Biosurfactant as a Promoter of Methane Hydrate Formation: Thermodynamic and Kinetic Studies
Source: Sci Rep. 2016 Feb 12;6:20893. doi: 10.1038/srep20893 (PMC4751436; doi:10.1038/srep20893)
Supplement: Supplementary Information [file srep20893-s1.pdf]

Biosurfactant as a Promoter of Methane Hydrate Formation: Thermodynamic and Kinetic Studies

Amit Arora*[1], Swaranjit Singh Cameotra[2], Rajnish Kumar[3], Chandrajit Balomajumder[1], Anil Kumar Singh[2], B. Santhakumari[4], Pushpendra Kumar[5], Sukumar Laik[6]

[1]Department of Chemical Engineering, Indian Institute of Technology, Roorkee, India., aroraamitlse@yahoo.com, chandfch@iitr.ernet.in

[2]Institute of Microbial Technology, Chandigarh, India, ssc@imtech.res.in, anilimtech@gmail.com

[3]Chemical Engineering and Process Development Division, National Chemical Laboratory, Pune, India. k.rajnish@ncl.res.in

[4] Centre For Material Characterization, National Chemical Laboratory, Pune, India. b.santhakumari@ncl.res.in

[5]Keshav Dev Malviya Institute of Petroleum Exploration, Oil and Natural Gas Corporation, Dehradun, India. pushpendrakumar_2005@yahoo.com

[6]Department of Petroleum Engineering, Indian School of Mines, Dhanbad, India. sukumar_ism@hotmail.com

***Corresponding author:** Amit Arora, Chemical Engineering Department, Indian Institute of Technology Roorkee, India,Roorkee -247667, India, E-mail: aroraamitlse@yahoo.com, +91-8427672776

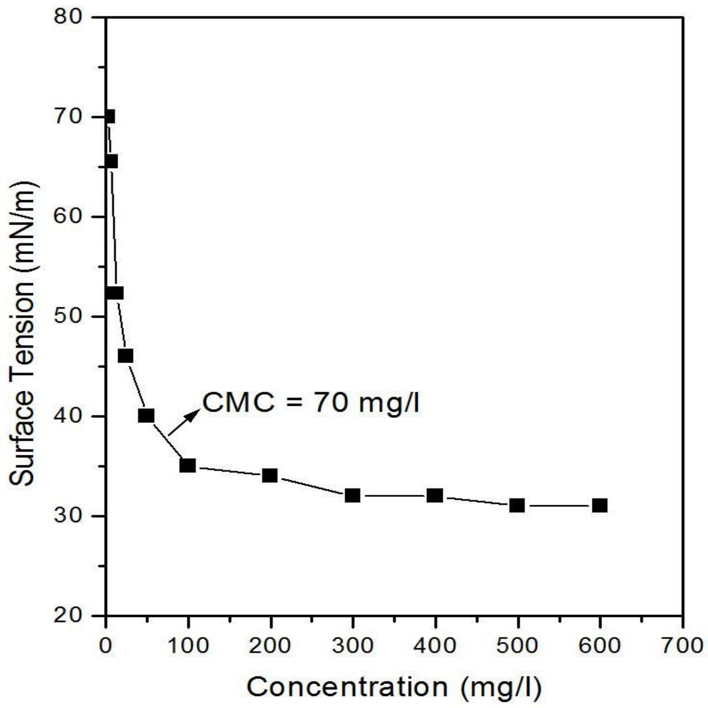

Supplementary Figure 1: Profile of surface tension reduction of different concentration of rhamnolipids in water. Arrow indicates the point of a sudden change in the surface tension and was designated as CMC. Values are mean of the results from three individual experiments

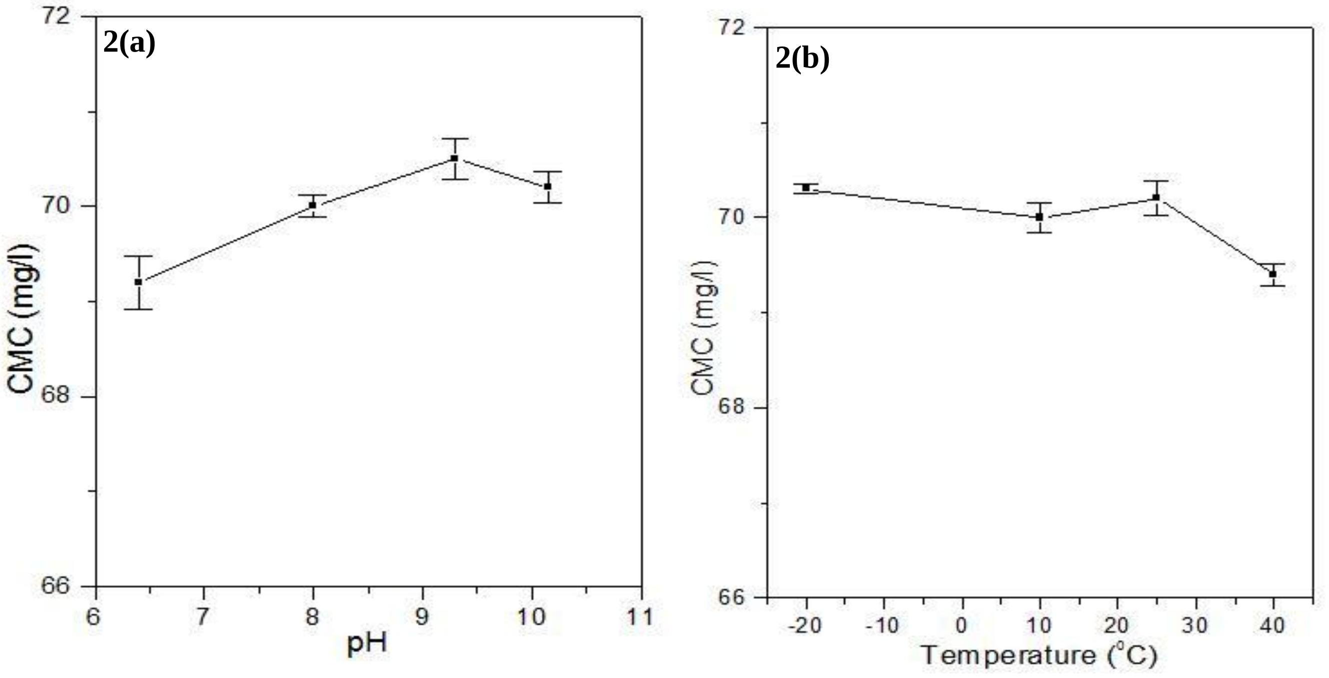

Supplementary Figure 2: Evaluation of rhamnolipids CMC at **(a)** different pH and **(b)** after challenging to different temperature. Values given are mean ± S.D. of three independent experiments.

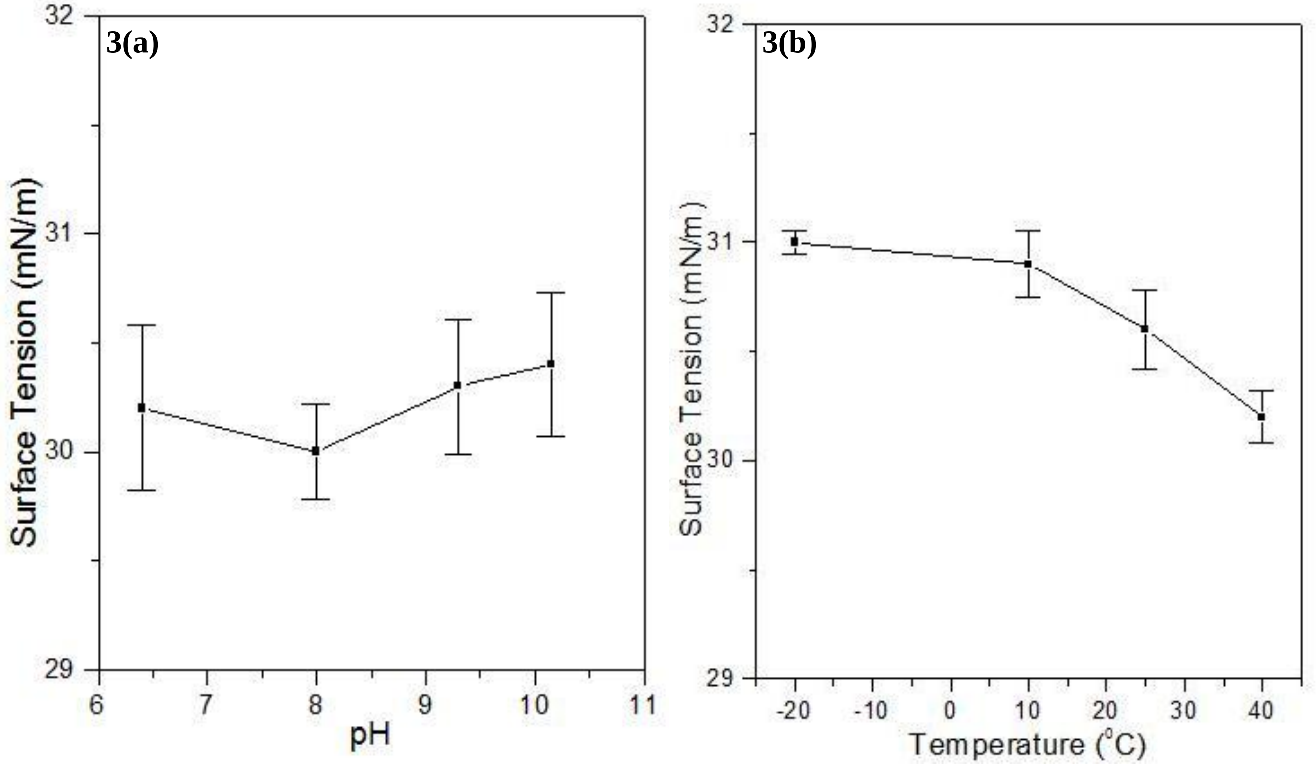

Supplementary Figure 3: Evaluation of rhamnolipids solution surface tension at **(a)** different pH and **(b)** after challenging to different temperature. Values given are mean ± S.D. of three independent experiments.

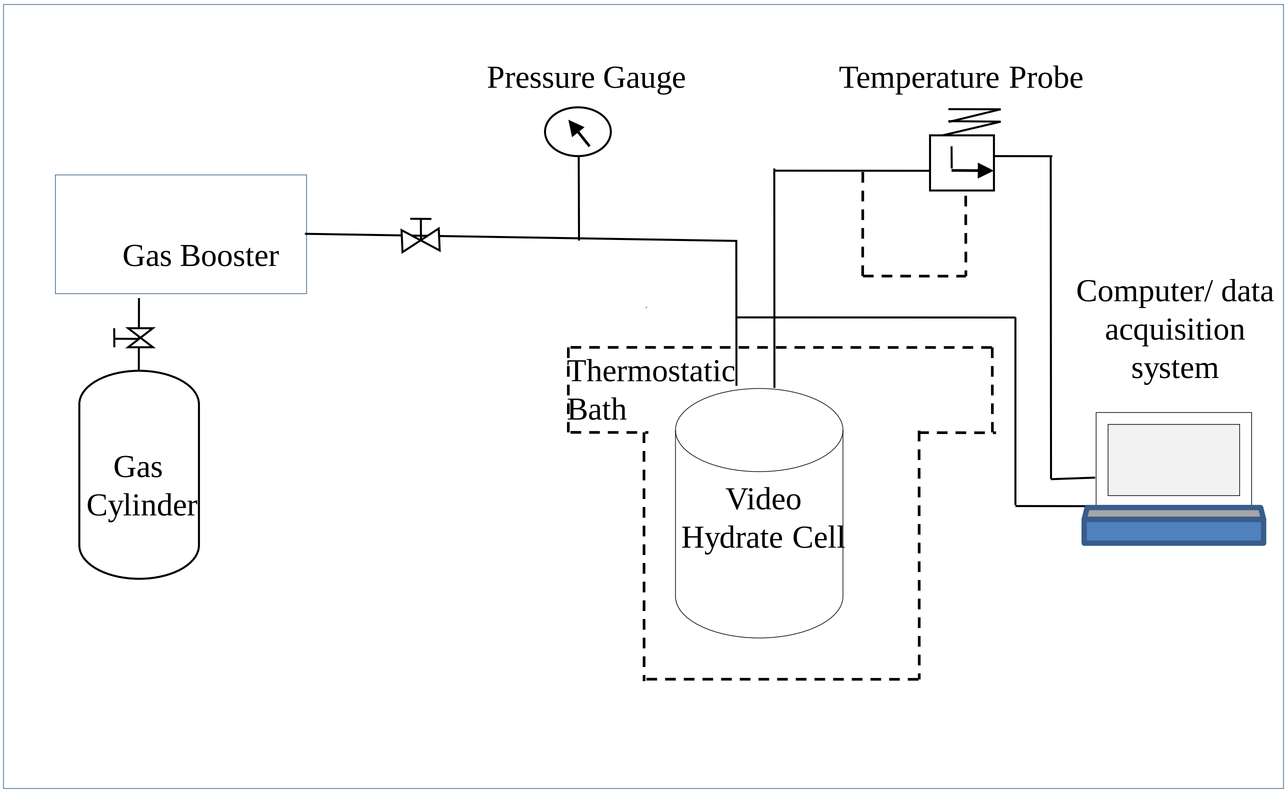

Supplementary Figure 4: Schematic diagram of the experimental apparatus
